# The Effect of Cellular Redox Status on the Evolvability of New Catabolic Pathways

**DOI:** 10.1128/mBio.01981-18

**Published:** 2018-10-16

**Authors:** Maia Kivisaar

**Affiliations:** a Institute of Molecular and Cell Biology, University of Tartu, Tartu, Estonia

**Keywords:** *Pseudomonas putida*, bacterial evolution, biodegradation, cellular redox status, mutation frequency, oxidative stress

## Abstract

Oxidation of aromatic compounds can be mutagenic due to the accumulation of reactive oxygen species (ROS) in bacterial cells and thereby facilitate evolution of corresponding catabolic pathways. To examine the effect of the background biochemical network on the evolvability of environmental bacteria hosting a new catabolic pathway, Akkaya and colleagues (mBio 9:e01512-18, 2018, https://doi.org/10.1128/mBio.01512-18) introduced the still-evolving 2,4-dinitrotoluene (2,4-DNT) pathway genes from the original environmental *Burkholderia* sp. isolate into the genome of Pseudomonas putida KT2440. They show that the mutagenic effect of 2,4-DNT oxidation, which is associated with the accumulation of ROS and oxidative damage on DNA, can be avoided by preserving high NADPH levels in P. putida. The observations of this study highlight the impact of the cellular redox status of bacteria on the evolvability of new metabolic pathways.

## Abstract (precis)

Oxidation of aromatic compounds can be mutagenic due to the accumulation of reactive oxygen species (ROS) in bacterial cells and thereby facilitate evolution of corresponding catabolic pathways. To examine the effect of the background biochemical network on the evolvability of environmental bacteria hosting a new catabolic pathway, Akkaya and colleagues (mBio 9:e01512-18, 2018, https://doi.org/10.1128/mBio.01512-18) introduced the still-evolving 2,4-dinitrotoluene (2,4-DNT) pathway genes from the original environmental *Burkholderia* sp.

## COMMENTARY

Catabolic pathways for naturally occurring, mostly plant-derived aromatic compounds are widely distributed in soil bacteria. Human-made xenobiotic compounds, in contrast, have been in the biosphere for only a few decades, but bacteria able to degrade such compounds have already been isolated. This demonstrates that under selective pressure exerted by pollutants, microbes can develop the capacity to degrade recalcitrant xenobiotics. For example, the ability to recognize and metabolize nitroaromatics by microorganisms might have been evolved only recently, since many nitroaromatic compounds are synthetic and have been introduced into the environment a short time ago (1). This process is still ongoing and therefore provides a good model for studying mechanisms of evolutionary processes in real time.

New catabolic pathways can evolve rapidly in bacteria as a result of horizontal gene transfer and point mutations that broaden the substrate range of preexisting enzymes (2–7). Mutational processes are the driving forces of evolution, and their rates fundamentally determine evolvability. The spontaneous mutation rate is generally held at a low level because most mutations are likely to be deleterious (8). However, bacterial populations with higher mutation rates can adapt to novel environments faster than those with lower mutation rates (9). Under certain circumstances, the frequency of mutations can be temporarily elevated. For example, accumulation of oxidative or alkylation damage in bacterial cells (10–12) and/or induction of the specific low-fidelity DNA polymerases when DNA is damaged can temporarily elevate the mutation rate (13, 14).

When present at a high concentration in cells, reactive oxygen species (ROS) have harmful effects on biological macromolecules such as proteins and nucleic acids (15). As mentioned above, oxidative damage to DNA is an important source of genetic variations (12). The classic strategy for the degradation of aromatic compounds comprises an attack by oxygenases that hydroxylate and finally cleave the aromatic ring with the help of activated molecular oxygen (16). Oxygenases can produce ROS by uncoupling of their catalytic mechanism (17). Moreover, it has been suggested that the ROS levels are further increased when oxygenases act on substrates that do not fit well in the active enzyme center (18). Thus, the evolution of new aerobic degradation pathways for xenoaromatic compounds could be associated with intracellular ROS generation, leading to elevated mutation frequency in cells.

A few years ago, Pérez-Pantoja and colleagues from Victor de Lorenzo’s laboratory reported that the first enzyme for the 2,4-dinitrotoluene (2,4-DNT) degradation pathway identified in *Burkholderia* sp. was not yet optimal for DNT degradation (7). Phylogenetic analysis of the *dnt* gene cluster encoding DNT biodegradation indicated that 2,4-DNT dioxygenase DntA, which catalyzes the initial oxidation of DNT, has been evolved from naphthalene dioxygenase (7, 19). However, the growth of bacteria on 2,4-DNT was associated with generation of high levels of ROS and an elevated mutation frequency (7). The production of ROS was associated with a faulty DNT dioxygenation reaction of DntA, which was no longer optimal for naphthalene nor entirely advantageous yet for DNT oxidation. Thus, the observations of this study provided a very good example of how the faulty dioxygenation reaction of the evolving enzyme elevates mutation frequency in the presence of new xenobiotic substrate and thereby accelerates evolution of the degradation pathway of this substrate.

In an article in *mBio*, Akkaya and colleagues (20) have further elucidated molecular mechanisms of evolution of xenobiotic degradation pathways by addressing the effect of the background biochemical network on the evolvability of environmental bacteria hosting a new catabolic pathway. The *dnt* genes encoding biodegradation of 2,4-DNT in *Burkholderia* sp. were introduced into the genome of a Pseudomonas putida KT2440 derivative which was previously designed for improved genetic stability and better heterologous gene expression (21–23). The effect of 2,4-DNT catabolism on intracellular ROS production, redox stress, and genetic variability was assessed in the engineered P. putida strain EM·DNT. It appeared that 2,4-DNT degradation resulted in ROS generation and activation of cellular response to oxidative stress. At the same time, the frequency of mutations was not significantly increased. This raised the question of what are the mechanisms by which P. putida avoids the increased rate of mutagenesis in the presence of ROS.

The answer may lie in high production of NADPH that protects/stabilizes the P. putida redox state. NADPH is an essential electron donor in all organisms. NADPH provides the reducing power that drives various anabolic reactions, including those responsible for the biosynthesis of all major cell components (24). NADPH is also necessary in providing reducing equivalents to regenerate antioxidative defense systems following ROS detoxification (25). For example, regeneration of reduced forms of glutathione and thioredoxin, which offer a first line of defense against ROS, utilizes NADPH as the cofactor. Observations made in the soil bacterium Pseudomonas fluorescens show that redirection of metabolic pathways toward routes that regenerate reducing power (e.g., NADPH) plays an important role in removal of ROS (26–28). Traditionally, the dehydrogenases directly coupled to central carbon metabolism (e.g., the oxidative pentose phosphate [PP] pathway, the Entner-Doudoroff [ED] pathway, and the isocitrate dehydrogenase step of the tricarboxylic acid [TCA] cycle) are involved in NADPH generation, but other NADPH-generating enzymes (e.g., transhydrogenases, ferredoxin NADP^+^ oxidoreductases, and NAD^+^ and NADH kinases) also play an important role in the redox homeostasis (24). P. putida KT2440 is a soil bacterium with a remarkable metabolic diversity, which enables it to degrade a wide variety of natural and recalcitrant aromatic compounds, whereas the presence of the ED pathway along with activities of the incomplete Embden-Meyerhof-Parnas (EMP) and PP pathways (EDEMP cycle) helps to counteract both exogenous and endogenous oxidative stress (29, 30). As the EDEMP cycle produces larger amounts of NADPH, it has been hypothesized that this provides an explanation of why pseudomonads are frequent hosts of operons that encode strong oxidative enzymes for biodegradation of aromatic pollutants (29, 31). Moreover, it was recently demonstrated that P. putida KT2440 encodes two nucleotide transhydrogenases that preserve the redox balance of bacteria during biodegradation of aromatic pollutants (32).

As the redox status of P. putida cells influences their sensitivity to ROS, which in turn could affect mutagenic processes, Akkaya et al. (20) decided to alter the redox status of bacteria in order to investigate the relationship between the redox status and the mutation frequency in P. putida EM·DNT. Indeed, the mutagenic effect of the 2,4-DNT degradation pathway was evident when the redox status of P. putida EM·DNT was artificially perturbed by overproducing an NADH oxidase (Nox) from Streptococcus pneumoniae. Furthermore, comparison of the spectrum of Rif^r^ mutations occurring in the *rpoB* gene revealed that the frequency of the occurrence of C-to-A transversions was significantly increased in the Nox-overexpressing P. putida cells in the presence of 2,4-DNT. 8-OxoG (GO) is known to be one of the most stable and frequent base modifications caused by oxygen radical attack on DNA (11). In order to mitigate the mutagenic effect of 8-oxoG, bacteria have developed an oxidized guanine (GO) repair system (33). The impairment of the GO repair system results in enhanced production of G·C-to-T·A transversions (34). Hence, the results from the work of Akkaya et al. (20) indicate that mutation rate can be affected by the endogenous redox status of the corresponding cells, whereas the increased mutagenesis in cells with decreased redox power is connected with DNA damage caused by ROS. Compared to the 2,4-DNT mutagenic effects observed in *Burkholderia* sp., the more reductive redox status in P. putida could provide effective protection against this mutagenic effect.

Taken together, this is an elegant study which demonstrates that the redox status of cells affects evolvability of P. putida toward novel xenobiotic substrates. In addition to biodegradation applications, P. putida is also employed as a cell factory in synthetic biology (for recent reviews, see, e.g., references 31, 35, and 36). From the work of Akkaya and colleagues, synthetic biologists can understand that achieving long-term stability of engineered producer strains requires cultivation of bacteria in a regime associated with high-level NADPH generation and ROS detoxification, whereas genetic diversification could be accelerated due to mutagenicity of ROS under conditions when NADPH becomes limiting. Thus, besides contributing to understanding mechanisms of evolutionary processes of new catabolic pathways, this knowledge might be important for bioengineering of P. putida with the purpose of bioproduction of value-added chemicals. Many natural products of industrial importance are complex secondary metabolites, the production of which often involves NADPH-dependent enzymes (24). As the synthesis of toxic chemicals could be associated with increased amounts of ROS and genetic instability of the engineered strains, the knowledge of connections between metabolism and evolvability of bacteria should be exploited for the rational design and operation of cell factories.

## References

[1] Ju KS, Parales RE 2010 Nitroaromatic compounds, from synthesis to biodegradation. Microbiol Mol Biol Rev 74:250–272. doi:10.1128/MMBR.00006-10. 20508249 PMC2884413

[2] Juhas M, van der Meer JR, Gaillard M, Harding RM, Hood DW, Crook DW 2009 Genomic islands: tools of bacterial horizontal gene transfer and evolution. FEMS Microbiol Rev 33:376–393. doi:10.1111/j.1574-6976.2008.00136.x. 19178566 PMC2704930

[3] Kivisaar M 2009 Degradation of nitroaromatic compounds: a model to study evolution of metabolic pathways. Mol Microbiol 74:777–781. doi:10.1111/j.1365-2958.2009.06905.x. 19818019

[4] Nojiri H, Shintani M, Omori T 2004 Divergence of mobile genetic elements involved in the distribution of xenobiotic-catabolic capacity. Appl Microbiol Biotechnol 64:154–174. doi:10.1007/s00253-003-1509-y. 14689248

[5] Norman A, Hansen LH, Sorensen SJ 2009 Conjugative plasmids: vessels of the communal gene pool. Philos Trans R Soc Lond B Biol Sci 364:2275–2289. doi:10.1098/rstb.2009.0037. 19571247 PMC2873005

[6] van der Meer JR, Sentchilo V 2003 Genomic islands and the evolution of catabolic pathways in bacteria. Curr Opin Biotechnol 14:248–254. doi:10.1016/S0958-1669(03)00058-2. 12849776

[7] Pérez-Pantoja D, Nikel PI, Chavarría M, de Lorenzo V 2013 Endogenous stress caused by faulty oxidation reactions fosters evolution of 2,4-dinitrotoluene-degrading bacteria. PLoS Genet 9:e1003764. doi:10.1371/journal.pgen.1003764. 24009532 PMC3757077

[8] Drake JW 1991 A constant rate of spontaneous mutation in DNA-based microbes. Proc Natl Acad Sci U S A 88:7160–7164. doi:10.1073/pnas.88.16.7160. 1831267 PMC52253

[9] Sniegowski PD, Gerrish PJ, Lenski RE 1997 Evolution of high mutation rates in experimental populations of *E. coli*. Nature 387:703–705. doi:10.1038/42701. 9192894

[10] Taverna P, Sedgwick B 1996 Generation of an endogenous DNA-methylating agent by nitrosation in *Escherichia coli*. J Bacteriol 178:5105–5111. doi:10.1128/jb.178.17.5105-5111.1996. 8752326 PMC178305

[11] Bjelland S, Seeberg E 2003 Mutagenicity, toxicity and repair of DNA base damage induced by oxidation. Mutat Res 531:37–80. doi:10.1016/j.mrfmmm.2003.07.002. 14637246

[12] Miller JH 1996 Spontaneous mutators in bacteria: insights into pathways of mutagenesis and repair. Annu Rev Microbiol 50:625–643. doi:10.1146/annurev.micro.50.1.625. 8905093

[13] Nohmi T 2006 Environmental stress and lesion-bypass DNA polymerases. Annu Rev Microbiol 60:231–253. doi:10.1146/annurev.micro.60.080805.142238. 16719715

[14] Ippoliti PJ, Delateur NA, Jones KM, Beuning PJ 2012 Multiple strategies for translesion synthesis in bacteria. Cells 1:799–831. doi:10.3390/cells1040799. 24710531 PMC3901139

[15] Imlay JA 2003 Pathways of oxidative damage. Annu Rev Microbiol 57:395–418. doi:10.1146/annurev.micro.57.030502.090938. 14527285

[16] Fuchs G, Boll M, Heider J 2011 Microbial degradation of aromatic compounds—from one strategy to four. Nat Rev Microbiol 9:803–816. doi:10.1038/nrmicro2652. 21963803

[17] Lee K 1999 Benzene-induced uncoupling of naphthalene dioxygenase activity and enzyme inactivation by production of hydrogen peroxide. J Bacteriol 181:2719–2725. 10217759 10.1128/jb.181.9.2719-2725.1999 PMC93710

[18] Gomez-Gil L, Kumar P, Barriault D, Bolin JT, Sylvestre M, Eltis LD 2007 Characterization of biphenyl dioxygenase of *Pandoraea pnomenusa* B-356 as a potent polychlorinated biphenyl-degrading enzyme. J Bacteriol 189:5705–5715. doi:10.1128/JB.01476-06. 17526697 PMC1951834

[19] Johnson GR, Jain RK, Spain JC 2002 Origins of the 2,4-dinitrotoluene pathway. J Bacteriol 184:4219–4232. doi:10.1128/JB.184.15.4219-4232.2002. 12107140 PMC135200

[20] Akkaya O, Perez-Pantoja DR, Calles B, Nikel PI, de Lorenzo V 2018 The metabolic redox regime of *Pseudomonas putida* tunes its evolvability toward novel xenobiotic substrates. mBio 9:e01512-18. doi:10.1128/mBio.01512-18. 30154264 PMC6113623

[21] Martinez-Garcia E, Nikel PI, Aparicio T, de Lorenzo V 2014 *Pseudomonas* 2.0: genetic upgrading of *P. putida* KT2440 as an enhanced host for heterologous gene expression. Microb Cell Fact 13:159. doi:10.1186/s12934-014-0159-3. 25384394 PMC4230525

[22] Martinez-Garcia E, Jatsenko T, Kivisaar M, de Lorenzo V 2015 Freeing Pseudomonas putida KT2440 of its proviral load strengthens endurance to environmental stresses. Environ Microbiol 17:76–90. doi:10.1111/1462-2920.12492. 24762028

[23] Lieder S, Nikel PI, de Lorenzo V, Takors R 2015 Genome reduction boosts heterologous gene expression in Pseudomonas putida. Microb Cell Fact 14:23. doi:10.1186/s12934-015-0207-7. 25890048 PMC4352270

[24] Spaans SK, Weusthuis RA, van der Oost J, Kengen SW 2015 NADPH-generating systems in bacteria and archaea. Front Microbiol 6:742. doi:10.3389/fmicb.2015.00742. 26284036 PMC4518329

[25] Lemire J, Alhasawi A, Appanna VP, Tharmalingam S, Appanna VD 2017 Metabolic defence against oxidative stress: the road less travelled so far. J Appl Microbiol 123:798–809. doi:10.1111/jam.13509. 28609580

[26] Singh R, Lemire J, Mailloux RJ, Appanna VD 2008 A novel strategy involved in [corrected] anti-oxidative defense: the conversion of NADH into NADPH by a metabolic network. PLoS One 3:e2682. doi:10.1371/annotation/5fac086b-3806-4aa9-a5c5-2611b3355f8f. 18628998 PMC2443280

[27] Singh R, Mailloux RJ, Puiseux-Dao S, Appanna VD 2007 Oxidative stress evokes a metabolic adaptation that favors increased NADPH synthesis and decreased NADH production in *Pseudomonas fluorescens*. J Bacteriol 189:6665–6675. doi:10.1128/JB.00555-07. 17573472 PMC2045160

[28] Mailloux RJ, Lemire J, Appanna VD 2011 Metabolic networks to combat oxidative stress in *Pseudomonas fluorescens*. Antonie Van Leeuwenhoek 99:433–442. doi:10.1007/s10482-010-9538-x. 21153706

[29] Chavarria M, Nikel PI, Perez-Pantoja D, de Lorenzo V 2013 The Entner-Doudoroff pathway empowers Pseudomonas putida KT2440 with a high tolerance to oxidative stress. Environ Microbiol 15:1772–1785. doi:10.1111/1462-2920.12069. 23301697

[30] Nikel PI, Chavarria M, Fuhrer T, Sauer U, de Lorenzo V 2015 *Pseudomonas putida* KT2440 strain metabolizes glucose through a cycle formed by enzymes of the Entner-Doudoroff, Embden-Meyerhof-Parnas, and pentose phosphate pathways. J Biol Chem 290:25920–25932. doi:10.1074/jbc.M115.687749. 26350459 PMC4646247

[31] Kim J, Salvador M, Saunders E, González J, Avignone-Rossa C, Jiménez JI 2016 Properties of alternative microbial hosts used in synthetic biology: towards the design of a modular chassis. Essays Biochem 60:303–313. doi:10.1042/EBC20160015. 27903818 PMC5264504

[32] Nikel PI, Perez-Pantoja D, de Lorenzo V 2016 Pyridine nucleotide transhydrogenases enable redox balance of *Pseudomonas putida* during biodegradation of aromatic compounds. Environ Microbiol 18:3565–3582. doi:10.1111/1462-2920.13434. 27348295

[33] Michaels ML, Cruz C, Grollman AP, Miller JH 1992 Evidence that MutY and MutM combine to prevent mutations by an oxidatively damaged form of guanine in DNA. Proc Natl Acad Sci U S A 89:7022–7025. doi:10.1073/pnas.89.15.7022. 1495996 PMC49637

[34] Michaels ML, Miller JH 1992 The GO system protects organisms from the mutagenic effect of the spontaneous lesion 8-hydroxyguanine (7,8-dihydro-8-oxoguanine). J Bacteriol 174:6321–6325. doi:10.1128/jb.174.20.6321-6325.1992. 1328155 PMC207574

[35] Calero P, Nikel PI 2018 Chasing bacterial chassis for metabolic engineering: a perspective review from classical to non-traditional microorganisms. Microb Biotechnol doi:10.1111/1751-7915.13292. PMC6302729 29926529

[36] Nikel PI, de Lorenzo V 2018 *Pseudomonas putida* as a functional chassis for industrial biocatalysis: from native biochemistry to trans-metabolism. Metab Eng doi:10.1016/j.ymben.2018.05.005. 29758287

